# Influence of the contact area of the sub-antral space with sinus bone and the Schneiderian membrane on osteogenesis in lateral window sinus elevation surgery: a prospective experiment

**DOI:** 10.1186/s12903-022-02694-1

**Published:** 2022-12-28

**Authors:** Xiao She, Dongjiao Zhang, Xin Xu, Zhanwei Zhang, Chonghao Ji, Zechuan Li, Dawei Song

**Affiliations:** 1 Department of Implantology, School and Hospital of Stomatology, Cheeloo College of Medicine, Shandong University & Shandong Key Laboratory of Oral Tissue Regeneration & Shandong Engineering Laboratory for Dental Materials and Oral Tissue Regeneration & Shandong Provincial Clinical Research Center for Oral Diseases, Jinan, China; 2 School of Stomatology, Shandong First Medical University and Shandong Academy of Medical Sciences, Jinan, 250117 China

**Keywords:** Bone regeneration, Clinical research, Maxillary sinus, CT imaging, Sinus floor elevation

## Abstract

**Background:**

Osteogenesis of lateral window sinus elevation surgery is the key to placement of the subsequent implant, excessive collapse of the sub-antral space may adversely affect long-term stability of implants. At present, few studies focus on the influence of the contact area of the sub-antral space on osteogenesis. This study evaluated whether the change in the contact area of the sub-antral space with maxillary sinus bone and the Schneiderian membrane can affect osteogenesis.

**Methods:**

Cone beam computed tomography (CBCT) images were collected of patients requiring maxillary sinus floor elevation (residual bone height < 6 mm) for standard-length implant placement before surgery, after surgery, and at 6-month follow-up visits. The postoperative sub-antral space volume (V_1_) and surface area (S_1_), and the remaining volume after six months of healing (V_2_) were measured. Then, the contact area of sub-antral space with maxillary sinus bone (S_bc_) and the Schneiderian membrane (S_mc_), the absorbed volume during healing (V_a_), and the percentage of remaining volume (V_2_%) and absorbed volume (V_a_%) were calculated. The correlation between anatomical parameters was analyzed using multiple linear regression.

**Results:**

A total of 62 maxillary sinuses from 56 patients were augmented, of which 57 were considered for the final analysis (5 withdrew due to perforation). Multiple linear regression results demonstrated that S_bc_ was significantly positively correlated with V_a_ (*β* coefficient = 0.141, *p* < 0.01) without correlation between S_mc_ and V_a_ (*β* coefficient =  − 0.046, *p* = 0.470). There was a positive correlation between S_bc_ and V_2_% (*β* coefficient = 2.269, *p* < 0.05).

**Conclusions:**

This study confirmed that the size of the S_bc_ in lateral window sinus elevation surgery affected osteogenesis after six months of healing. Clinicians should assess the sinus contour type preoperatively, then consider whether it is necessary to expand the range of the Schneiderian membrane elevation to avoid excessive collapse of the sub-antral space.

*Trial registration*: Chinese Clinical Trial Registry (ChiCTR), ChiCTR2200057924. Registered 22 March 2022–Retrospectively registered.

## Background

Lateral window sinus elevation surgery was first presented by Tatum in 1976 and then published by Boyne and James in 1980. It is an innovative method that can predictably increase the height available at the bone in the maxillary posterior tooth area to place a standard-length implant [1]. The best material for this surgery is autologous bone due to its excellent bone conduction, bone induction, and osteogenesis [2]. Although the new bone formation rate of autologous bone is significantly higher than that of other types of bone graft materials, it also has disadvantages, such as the need for a second surgical site, the limited amount, and low dimensional stability [3]. In particular, low dimensional stability means the absorption of graft materials and the collapse of the graft volume, which can significantly affect surgical outcomes [4, 5]. Therefore, new graft materials have been developed, including allografts and xenografts, and their applications in clinical treatment and scientific research have been studied in-depth [6–9]. Xenografts are widely used due to their high three-dimensional stability during healing after bone augmentation surgery [10, 11].

During the healing process after lateral window sinus elevation surgery, the two major biological behaviors affecting osteogenesis are extensive neoangiogenesis and the migration and colonization of osteoprogenitor cells from surrounding bone [12, 13]. The Schneiderian membrane and the maxillary sinus bone support this physiological process, but the Schneiderian membrane does not consistently significantly contribute to new bone formation [14]. Some animal experiments depict that the pluripotent mesenchymal cells of the Schneiderian membrane contribute to osteogenesis [15, 16], while other animal experiments suggested no obvious effect of the Schneiderian membrane on new bone formation. However, the effect of sinus bone on osteogenesis has been widely recognized in many studies [17, 18]. So how do the Schneiderian membrane and maxillary sinus bone affect those biological processes of osteogenesis after sinus augmentation? The size of S_bc_ and S_mc_ may significantly impact osteogenesis since better bone formation was observed when the diameter of bone graft material particles decreased to give a larger contact area in an in vitro tissue engineering study [19]. In maxillary sinuses with different sinus widths, the contact area between the implant and sinus walls is larger in the narrow sinus, which promotes the vascular blood supply for bone formation [20]. Nevertheless, to date, there is little research focusing on the influence of S_bc_ and S_mc_ on the osteogenic effect. Therefore, this prospective study aimed to determine how S_bc_ and S_mc_ affect osteogenesis following lateral window sinus elevation surgery.

## Methods

### Study protocol

This prospective cohort study was reported according to the STROBE statement. All procedures were conducted according to the guidelines of the Declaration of Helsinki as revised in Fortaleza (2013) for human subject research. The study protocol was approved by the ethics committee of Stomatology Hospital, Shandong University, China (No. 20190107), and registered in the Clinical Trial Registry (ChiCTR2200057924). The patients provided written informed consent to participate in the trial and authorized the use of their data for the study purposes after being informed of the study protocol, treatment protocol, alternatives, and any potential dangers.

### Selection criteria

Any patient with missing teeth in the maxillary posterior area and requiring lateral window sinus elevation surgery to increase the available bone height to place a standard-length implant was eligible for inclusion. The inclusion criteria were: age > 18 years; edentulism in the maxillary premolar and molar region for at least three months; residual bone height (RBH) < 6 mm and residual bone crest width ≥ 6 mm in site(s) prepared for the implant placement; systemic condition sufficient to undergo surgery; willing to provide informed consent, good medical compliance, and be able to return to the hospital regularly. The exclusion criteria were: absolute contraindications for implant surgery or bone augmentation surgery [21]; smokers (≥ 10 cigarettes/day); pregnancy or lactation; uncontrolled systemic diseases (diabetes, hypertension, autoimmune diseases, etc.); undergoing head and neck radiotherapy or bisphosphate treatment; allergic to materials that they may be exposed to during the operation; uncontrolled periodontal disease, dental pulp disease or other oral diseases; suffering from maxillary sinusitis (thickness of the Schneiderian membrane > 2 mm, sinus effusion, and sinus density increased) [22–24]; previous implant or bone augmentation surgery at the surgical site; the Schneiderian membrane perforation during the operation.

### Pre-surgical phase

Comprehensive medical history collection and clinical examination were conducted for the patients included in this study. The oral examination included a periodontal and endodontic examination and CBCT evaluation of the health status of the sinus and the residual bone quantity at the planned implant site. The patients were divided into two groups according to the RBH based on expert consensus and ITI treatment guide of bone augmentation surgery [25, 26]: group A (3 mm ≤ RBH < 6 mm): simultaneous implant group, and group B (RBH < 3 mm): delayed implant group (Table 1).

**Table 1:** Baseline data of patients in group A and B

	Number	A	B
*Gender*			
Male	25	14	10
Female	26	16	11
Age (Mean ± SD)	51.8 ± 11.9	51.2 ± 12.4	52.4 ± 11.3
*Number of missing teeth*			
1	18	10	8
2	23	14	9
3	10	6	4
*Implant length*			
10 mm	33	19	14
12 mm	18	11	7
*Implant length*			
4.1 mm	28	16	12
4.8 mm	23	14	9

### Surgical procedure

**Fig. 1:**
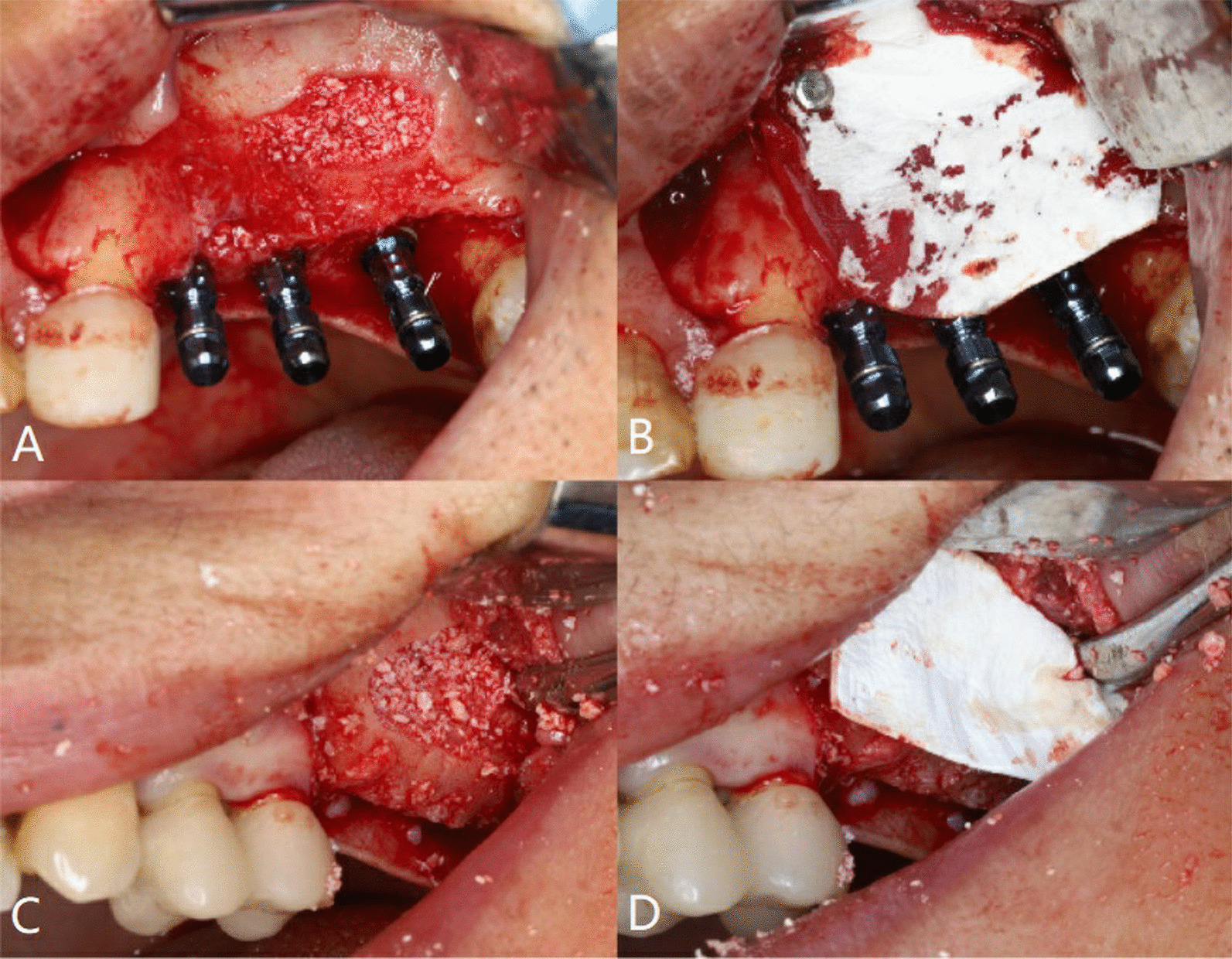
Intraoperative photos. **A** and **B** from group A: Simultaneous implantation after completing sinus elevation; **C** and **D** from group B: Only sinus elevation was completed during the first surgery

Antibiotics (cefixime, metronidazole) were administered 0.5 h before the operation to prevent infection, and the mouths were rinsed with compound chlorhexidine mouthwash three times for 30 s. Concentrated growth factor (CGF) was prepared as instructed 10 min before surgery [27]. Under local anesthesia, a mid-crestal incision and two buccal releasing incisions were made, and the full-thickness flap was lifted to expose the buccal bone wall of the sinus. After removing the lateral wall, the Schneiderian membrane was carefully and evenly elevated with manual instruments until the sub-antral space reached the preset size. After checking the integrity of the Schneiderian membrane by visual inspection and the Valsalva maneuver, two to four CGF membranes were inserted into the sub-antral space, the mixture of red blood cells/platelets, and deproteinized bovine bone matrix (Bio-Oss®, Geistlich AG, Switzerland) was then grafted. A gelatin sponge was used to stop bleeding if necessary. A resorbable bovine collagen membrane (Bio-Gide®, Geistlich AG, Switzerland) was secured with pins to cover the lateral antrostomy. The patients in group A received 10–12 mm long implants (Straumann AG, Switzerland), and the flap was sutured with non-absorbable surgical sutures (Fig. 1). Postoperative CBCT images were taken immediately. Each patient was given antibiotics (Cefixime, metronidazole) for three days, gargled with compound chlorhexidine for one week, and given acetaminophen if necessary. The precautions after the operation were explained, and patients were informed to remove the sutures ten days later. The healing status of the patients was checked monthly.

After six months of healing, patients returned to the hospital for CBCT images to evaluate recovery. Patients in group B received 10–12 mm long implants (Straumann AG, Switzerland). The patients in both groups continued to fix superstructure restoration. The CBCT images collected from each patient included T_0_ (preoperative), T_1_ (immediately after operation), and T_2_ (six months after operation).

### Radiographic measurements

**Fig. 2:**
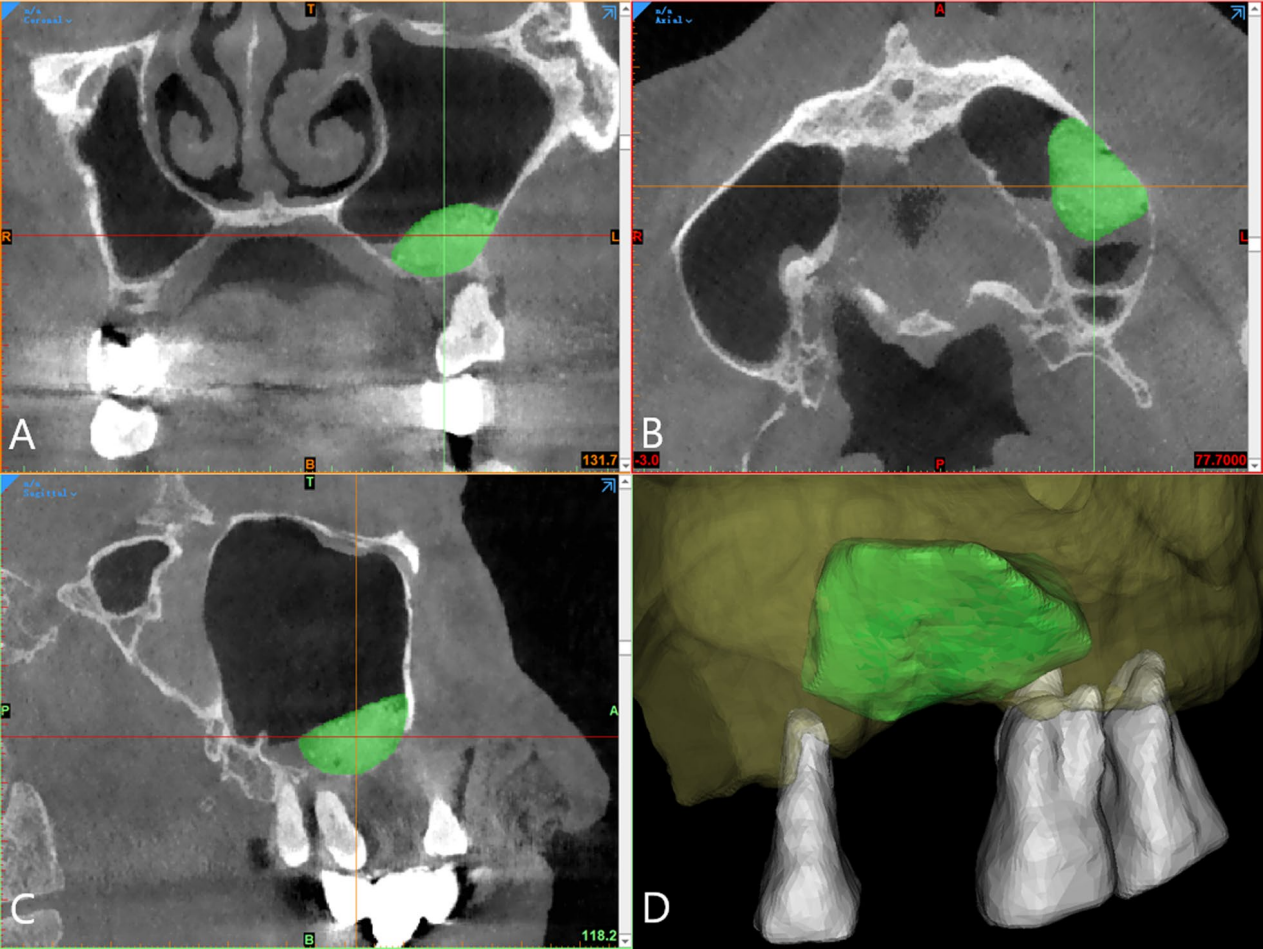
Volume and surface area analysis was performed using Mimics. **A** “Green mask” of the sub-antral space was created as the region of interest. **A**–**C**: the coronal, axial and sagittal planes of sub-antral space. **D**: A 3-dimensional model, including sub-antral space (green), bone tissue around maxillary sinus (yellow) and some nearby teeth (white)

**Fig. 3:**
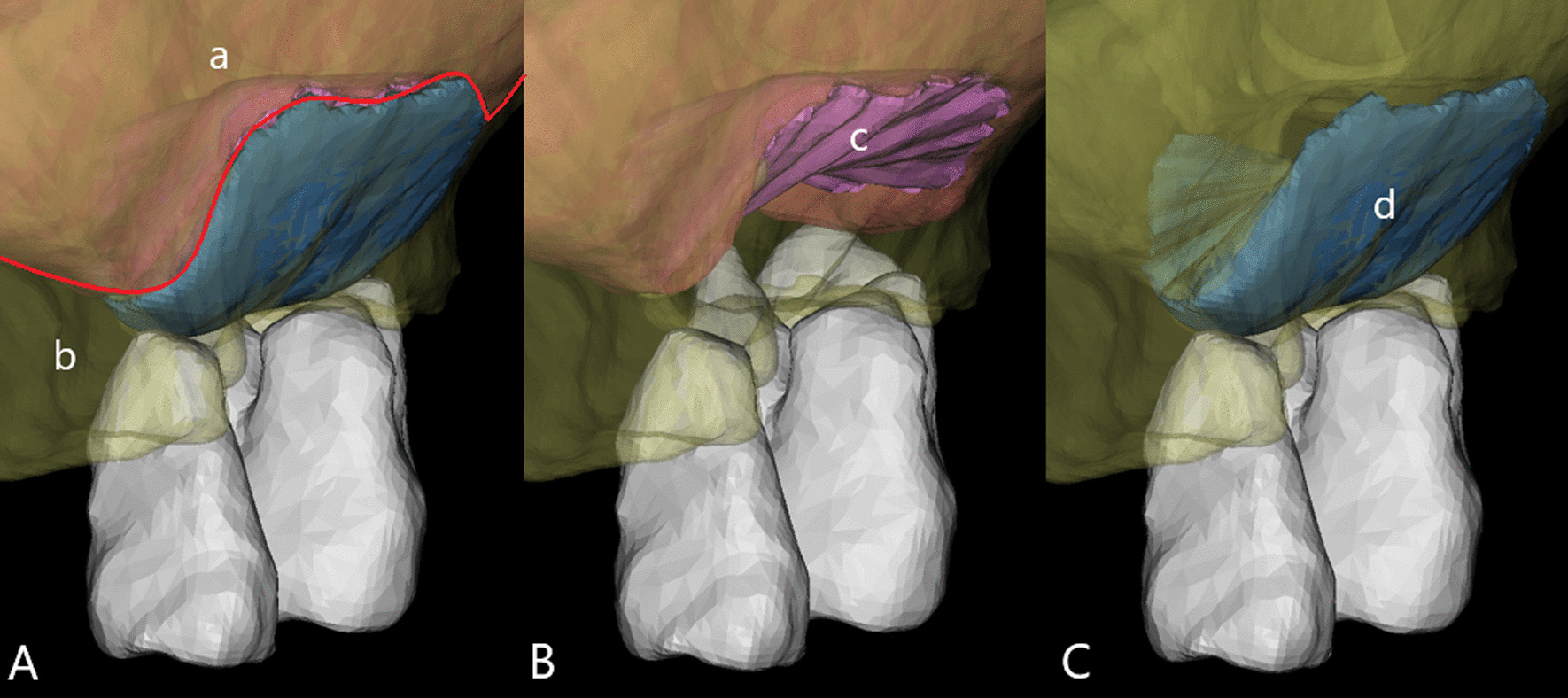
Contact area analysis using Mimics. **A** Sub-antral space was divided into two parts according to its contact relationship with maxillary sinus bone wall and the Schneiderian membrane, (**a**) the Schneiderian membrane, the red line marks its lower boundary, (**b**) maxillary sinus bone. **B** The sub-antral space that in contact with the Schneiderian membrane (**c**). **C** the sub-antral space that in contact with maxillary sinus bone (**d**)

**Fig. 4:**
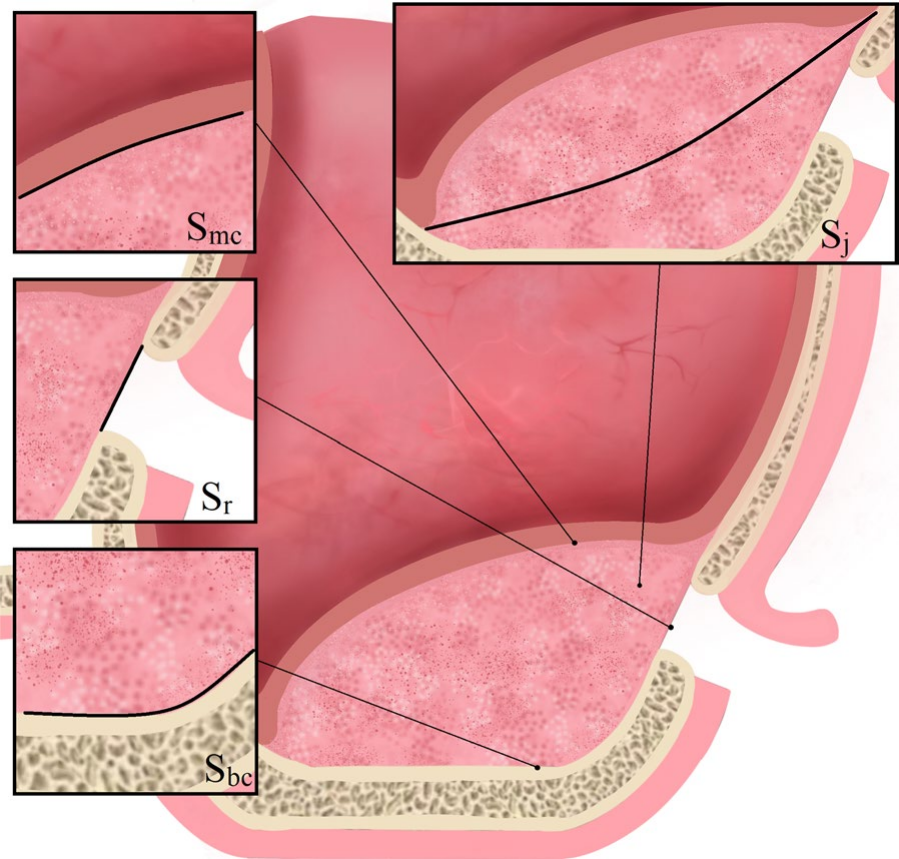
Contact relationship between sub-antral space and surrounding tissue. S_mc_: the contact area of sub-antral space with schneiderian membrane. S_r_: the surface area of the removed lateral bone wall. S_bc_: the contact area of sub-antral space with maxillary sinus bone. S_j_: the surface area of an imaginary plane, which divides the sub-antral space into upper and lower parts, the upper part contacts the Schneiderian membrane, while the lower part contacts the sinus bone

The data were analyzed by three independent surveyors using image processing software (Mimics Medical version 21.0). After confirming that there were no size errors, the sub-antral space was divided to check the coronal, axial, and sagittal planes for deviation before the 3D reconstruction of the model (Fig. 2). Automatically calculate the basic data of sub-antral space according to those models, including the postoperative volume (V_1_), remaining volume after six months of healing (V_2_) and surface area at T_1_ (S_1_). The sub-antral space at T_1_ was divided into two parts according to the contact relationship with the maxillary sinus bone wall and the Schneiderian membrane (Fig. 3); the surface area was automatically calculated as S_b_ (surface area of the sub-antral space in contact with bone) and S_m_ (surface area of the sub-antral space in contact with the Schneiderian membrane) respectively. The S_j_ was defined as the surface area of an imaginary plane, which divides the sub-antral space into upper and lower parts; the upper part contacts the Schneiderian membrane, while the lower part contacts the sinus bone (S_j_ is only used as the transition value for calculating S_bc_ and S_mc_, and how the imaginary plane divides the sub-antral space does not affect the calculation of subsequent values). The surface area of the removed lateral bone wall was defined as S_r_. For group A, the volume of the implant entering the sub-antral space should be subtracted when calculating V_1_ and V_2_, and the cross-sectional area of the implant should be subtracted when calculating S_bc_. The surface areas and their relationships are illustrated in Fig. 4.

### Numeral calculations

Some of the values used in the statistical analysis were calculated according to the following formulas:

$$\begin{aligned} {\text{V}}_{{\text{a}}} & = {\text{V}}_{2} - {\text{V}}_{1} \\ {\text{V}}_{{\text{a}}} \% & = \frac{{{\text{V}}_{{\text{a}}} }}{{{\text{V}}_{1} }} \times 100\% \\ {\text{V}}_{{2}} \% & = \frac{{{\text{V}}_{{2}} }}{{{\text{V}}_{1} }} \times 100\% \\ {\text{S}}_{{\text{j}}} & = \frac{{\left\{ {\left( {{\text{S}}_{{\text{b}}} + {\text{S}}_{{\text{m}}} } \right) - {\text{S}}_{1} } \right\}}}{2} \\ {\text{S}}_{{{\text{bc}}}} & = {\text{S}}_{{\text{b}}} - {\text{S}}_{{\text{j}}} - {\text{S}}_{{\text{r}}} \\ {\text{S}}_{{{\text{mc}}}} & = {\text{S}}_{{\text{m}}} - {\text{S}}_{{\text{j}}} \\ \end{aligned}$$

### Predictor and outcome variables

The null hypothesis in this prospective research was that the change in the contact area of the sub-antral space with bone and the Schneiderian membrane does not affect osteogenesis. The primary predictor variables are the contact area of sub-antral space with bone (S_bc_) and the Schneiderian membrane (S_mc_).

Primary outcome measure: the absorbed volume of the sub-antral space (V_a_).

Secondary outcome measures: the remaining volume of the sub-antral space (V_2_); percentage of the absorbed volume (V_a_%); percentage of the remaining volume (V_2_%); the occurrence of any complications.

### Statistical analysis

Data were collected by an independent investigator and analyzed using statistical software (IBM SPSS Statistics for Windows, Version 26.0, IBM Corp.). The intraclass correlation coefficient (ICC) was used to compare the reproducibility between the three surveyors. The Shapiro–Wilk test was used to evaluate the existence of normal distribution, the assumptions required to apply the parameter test are met by all parameters. Descriptive statistics were fully recorded, including mean and standard deviation. Independent sample t-tests were performed to evaluate the difference between V_a_, V_2_, V_a_%, and V_2_% in groups A and B. Multiple linear regression analysis was performed to study the influence of V_a_, V_2_, V_a_%, and V_2_%, respectively, and the independent factors were V_1_, S_bc,_ and S_mc_. Durbin-Watson test was used to evaluate the independence of samples, with R-squared values as a goodness-of-fit measure. A *p-value* < 0.5 was considered statistically significant.

## Results

### Study population and clinical results

The study initially included 62 maxillary sinuses from 56 patients, all of whom underwent lateral window sinus elevation surgery in the Department of Oral Implantology, Stomatology Hospital, Shandong University, Jinan, China, from January 2019 to August 2021. The surgeries were performed by three experienced implantologists. Due to the different sizes of the Schneiderian membrane perforations during surgery, five patients withdrew from the study, and after perforation repair, those patients did not suffer serious complications during the follow-up period of at least one year. Ultimately, 57 maxillary sinuses from 51 patients (25 males; 26 females; mean age 51.8 ± 11.9; age range 22–74 years) were analyzed: 33 cases in group A (simultaneous implant) and 24 in group B (delayed implant). During the follow-up period of 7–38 months, all implants functioned normally, without complications or adverse events during the intraoperative, postoperative, and follow-up phases.

### Radiographic measurements

The mean measured values of the three surveyors were analyzed and were highly consistent (ICC > 0.830). Table 2 presents the volume of sub-antral space in groups A and B immediately after the operation and after six months of healing, showing no significant difference in bone formation between the two groups. Therefore, in the subsequent analysis, the two data groups were combined into one group.

**Table 2:** Comparison of the bone formation of the two groups A and B

	A		B		*p*-value
	Mean ± SD	Range	Mean ± SD	Range	
V_1_ (cm^3^)	1.55 ± 0.85	0.35–4.88	1.54 ± 0.77	0.48–3.46	0.977
V_2_ (cm^3^)	1.19 ± 0.62	0.21–3.11	1.24 ± 0.71	0.33–3.11	0.789
V_a_ (cm^3^)	0.36 ± 0.33	0.01–1.76	0.30 ± 0.22	0.07–0.95	0.491
V_2_%	(77.31 ± 11.28)%	(60.13–97.26)%	(78.53 ± 12.97)%	(42.38–95.88)%	0.705
V_a_%	(22.69 ± 11.28)%	(2.74–39.87)%	(21.47 ± 12.97)%	(4.12–57.62)%	0.705

SD, Standard deviation; V_1_, volume of postoperative sub-antral space; V_2_, volume of sub-antral space 6 months after healing; V_a_, volume of absorbed during 6 months of healing; V_2_%, percentage of remaining volume in V_1_; V_a_%, percentage of absorbed volume in V_1_

As displayed in Table 3, linear regression analysis indicated a significant positive correlation between V_1_ and V_a_ (*p* < 0.001). Table 4 illustrates the linear regression results for the contact area (S_bc_ and S_mc_) and V_a_, revealing a significant positive correlation between S_bc_ and V_a_ (*p* = 0.002) but no correlation between S_mc_ and V_a_ (*p* = 0.470). This suggests that S_bc_ is more likely than S_mc_ to affect V_a_; thus, we focused on S_bc_ in the following analysis. Table 5 depicts that S_bc_ and V_2_ are significantly positively correlated (*p* < 0.001).

**Table 3:** Linear regression analysis of the effect of V_1_ on V_a_

	*β* coefficient	SE	t	*p*-value
Model: Outcome V_a_ ${\overline{\text{R}}}^{{2}} = 0.{892}$				
V_1_	0.764	0.036	21.260	< 0.001

SE, Standardized error of the β coefficient; t, t-value; V_1_, volume of postoperative sub-antral space; V_a_, volume of absorbed during 6 months of healing

**Table 4:** Multiple linear regression was used to detect the factors affecting V_a_

	*β* coefficient	SE	t	*p*-value
Model: outcome V_a_ ${\overline{\text{R}}}^{{2}} = 0.{397}$				
S_bc_	0.141	0.044	3.220	0.002
S_mc_	− 0.046	0.063	− 0.728	0.470

SE, Standardized error of the β coefficient; t, t-value; S_bc_, contact area between sub-antral space and bone; S_mc_, contact area between sub-antral space and the Schneiderian membrane

**Table 5:** Linear regression analysis of the effect of S_bc_ on V_2_

	*β* coefficient	SE	t	*p*-value
Model: outcome V_a_ ${\overline{\text{R}}}^{{2}} = 0.{859}$				
S_bc_	0.378	0.021	18.327	< 0.001

SE, Standardized error of the β coefficient; t, t-value; S_bc_, contact area between sub-antral space and bone

Tables 6 and 7 displays the linear regression results of volume percentage (V_a_% and V_2_%) and S_bc_, indicating a significantly positive correlation between S_bc_ and V_2_% and a significantly negative correlation between S_bc_ and V_a_%. Since the sum of V_a_% and V_2_% is 1, the two tables are equal except for the opposite *β* coefficient value.

**Table 6:** Linear regression analysis of the effect of S_bc_ on V_a_%

Association between S_bc_ and V_a_%				
	*β* coefficient	SE	t	*p*-value
Model: outcome V_a_% ${\overline{\text{R}}}^{{2}} = 0.{074}$				
S_bc_	− 2.269	1.104	− 2.056	0.045

SE, Standardized error of the β coefficient; t, t-value; S_bc_, contact area between sub-antral space and bone; V_a_%, percentage of absorbed volume in V_1_

**Table 7:** Linear regression analysis of the effect of S_bc_ on V_2_%

Association between S_bc_ and V_2_%				
	*β* coefficient	SE	t	*p*-value
Model: outcome V_2_% ${\overline{\text{R}}}^{{2}} = 0.{074}$				
S_bc_	2.269	1.104	2.056	0.045

SE, Standardized error of the β coefficient; t, t-value; S_bc_, contact area between sub-antral space and bone; V_2_%, percentage of remaining volume in V_1_

## Discussion

The success of maxillary sinus floor elevation was due to the effect of new bone formation, which is influenced by anatomical morphology, bone graft substitutes, surgical methods, etc. In the process of new bone formation, extensive neovascularization, migration, and colonization of bone progenitor cells are two key biological steps of postoperative healing. The neovascular network and osteoprogenitor cells may come from the bone or sinus membrane in contact with the graft materials. This signifies that the size of the contact area is likely to affect osteogenesis; however, this is little literature regarding its impact, how it affects, and the specific impact relationship.

**Fig. 5:**
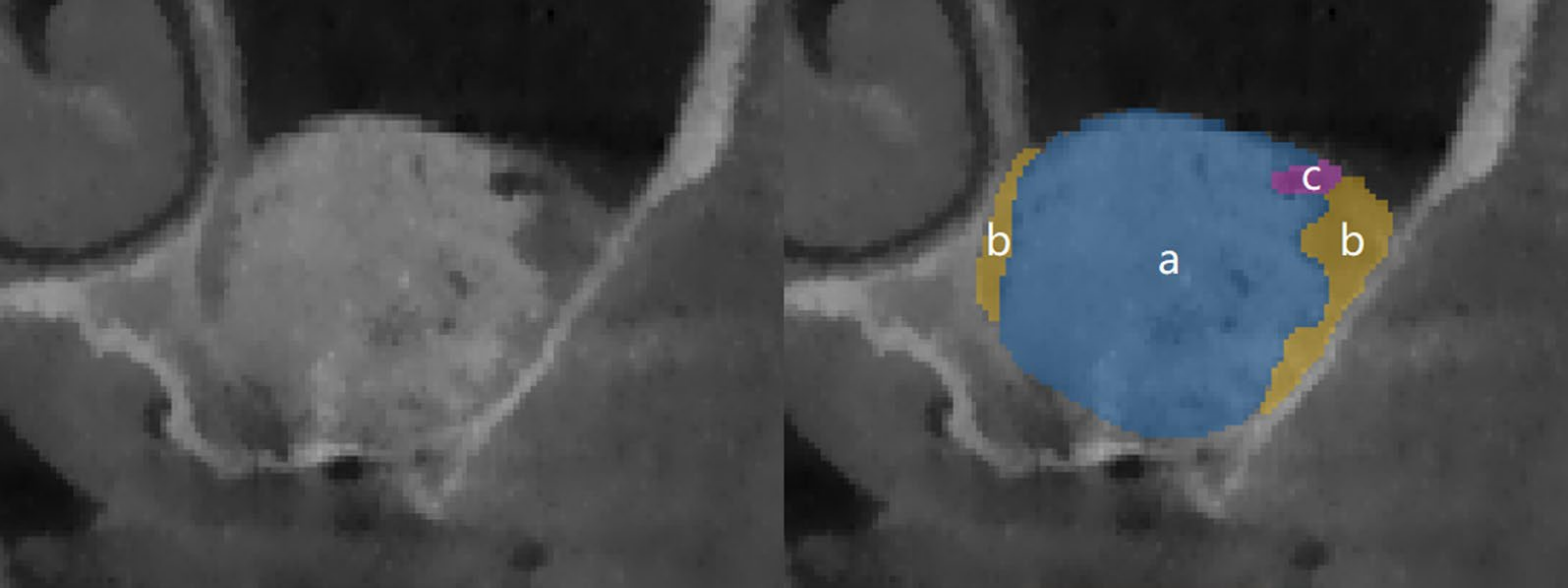
Contents of sub-antral space. **a** Bio-oss, **b** CGF and blood, **c** air

This study evaluated the effect of S_bc_ and S_mc_ on Va, showing that the graft material is in contact with the maxillary sinus bone, the more it will be absorbed, and for every 1 cm^2^ increase in the contact area, the absorbed volume increases by 0.141 cm^3^. During the healing process, the sub-antral space volume reduction is mainly due to the absorption of the contents, which in addition to the bone substitute, contains CGF, blood, and air (Fig. 5), but the metabolism rate of these contents is much faster than that of the bone substitute. Therefore, when the S_bc_ is larger, these contents will be absorbed faster, and the Schneiderian membrane does not participate in this absorption process, which indicates that the contribution of bone to the absorption of these contents is much greater than that of the Schneiderian membrane. Although the bone material will also be absorbed during the healing, since Bio-oss is a long-term degradation material that is difficult to be absorbed [28], it can exist in the body for a long time [29], so the volume of this part is negligible. Second, the reduction of sub-antral space volume is also related to the collapse of the three-dimensional structure of bone material, which may be affected by the negative pressure generated by the absorption of contents within the gaps of bone material particles, and the process is associated with the extra pressure on the Schneiderian membrane [5, 30], which is unavoidable due to respiration [31]. However, no effect of S_mc_ on V_a_ was observed in this study, so the effect of pressure from breathing on collapse may be much greater than that of S_mc_.

This study analyzed the effect of S_bc_ on V_2,_ showing that there is a considerable positive association between S_bc_ and V_2_, illustrating the relationship between S_bc_ and the osteogenic effect. The larger S_bc_ can preserve more bone graft after six months of healing. This argument resembles the one mentioned in the previous paragraph that "the larger the S_bc_, the greater the amount of absorbed graft material", which is the correlationship between volume and area. However, there is often a strong inherent correlation between the volume and area values of sub-antral space; in other words, the larger the volume, the larger the area, and vice versa. So, the significant positive correlation between S_bc_ and V_2_ may not fully explain the influence of S_bc_ on osteogenesis. Therefore, ratios were used instead of numerical values to represent the osteogenic effect to avoid the influence of the strong correlation between volume and area on the results. S_bc_ was significantly positively correlated with V_2_% and significantly negatively correlated with V_a_%, and when S_bc_ increased by 1 cm^2^, V_2_% increased by 2.27%. This indicates that the larger the S_bc_, the higher the volume of graft material maintained, and the more new bone can be obtained. The possible reason for this, as mentioned earlier, is that a larger contact area leads to more osteoprogenitor cell migration, as well as a wider range of neovascularization.

**Fig. 6:**
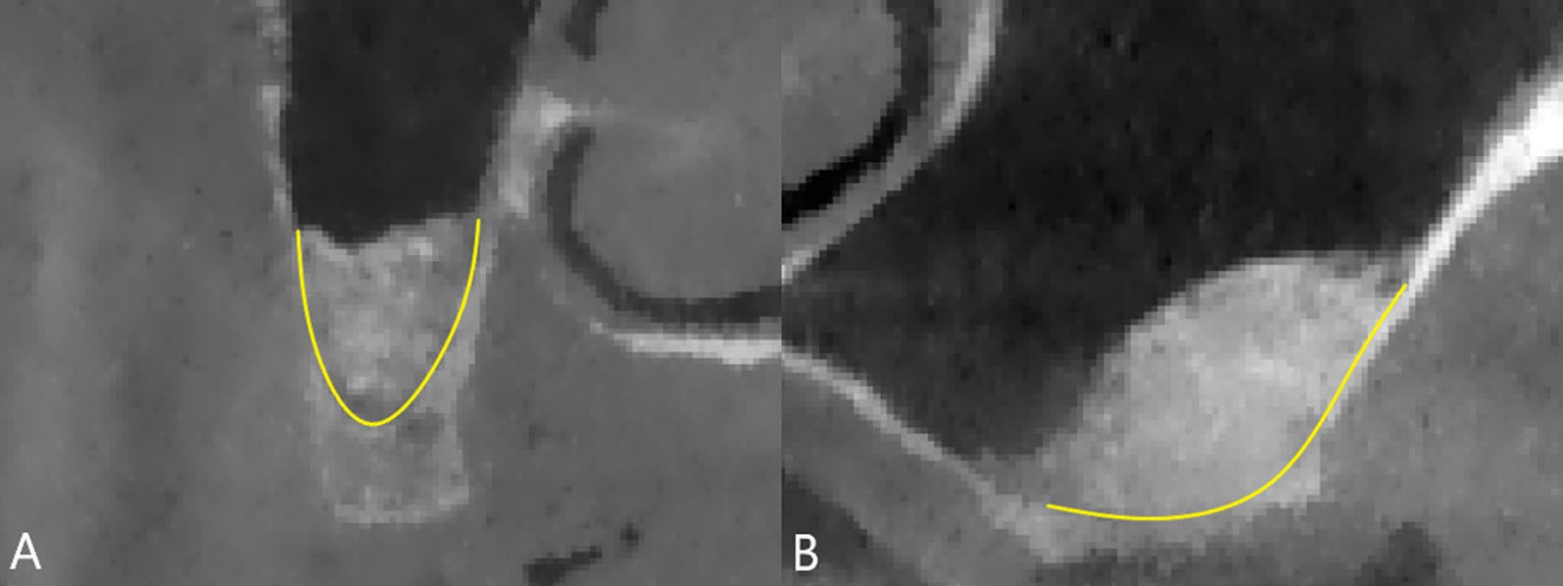
Comparison of the contact area between sub-antral space and sinus bone after sinus membrane elevation for maxillary sinus with different contours. Yellow line represents the contact area. **A**: narrow tapered, **B**: square

**Fig. 7:**
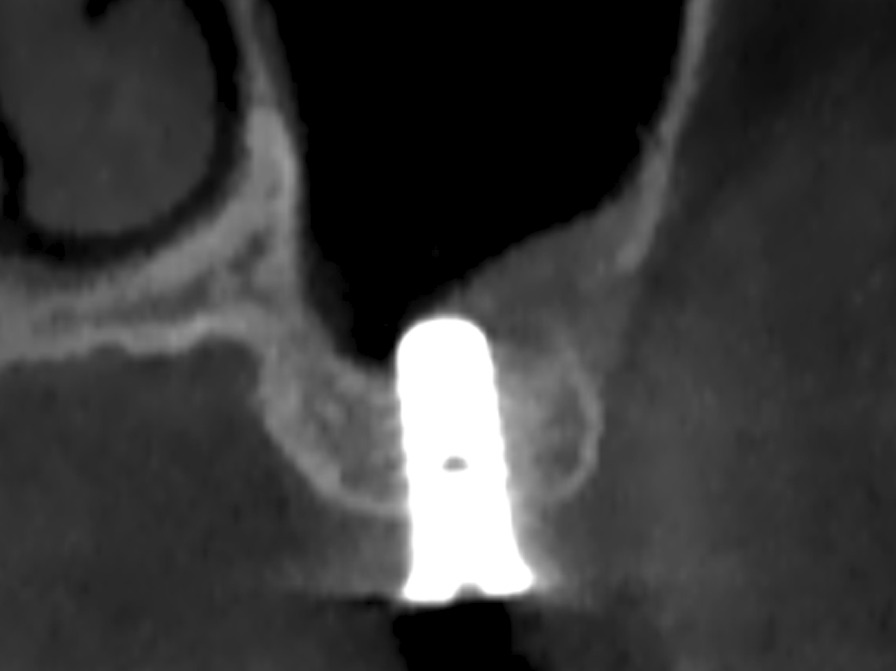
The implant is in contact with the sinus membrane due to the absorption of graft material

Significant correlation of S_bc_ with V_2_% and V_a_% has important implications for clinical and research work. Clinicians not only need to consider the patient factors (systemic condition, oral condition, maxillary sinus anatomy, etc.), select the appropriate type of graft material and dosage, but appropriately increase the contact area of the graft material with the sinus wall and sinus floor during the operation when faced with a patient who may require sinus bone augmentation to gain more neogenetic bone. In the light of the classification of maxillary sinus contours [32], in the narrow tapered maxillary sinus, the proper elevation of the maxillary sinus membrane and filling of graft materials can achieve a large contact area between the sub-antral space and the bone, resulting in a good osteogenic effect (Fig. 6A). However, in the square maxillary sinus, especially at sites that require delayed implantation, the traditional sinus membrane elevation range often cannot obtain a sufficient S_bc_, so it is necessary to consider continuing to elevate the sinus membrane (Fig. 6B); otherwise, the bone height after six months of healing may be lower than expected. In square maxillary sinuses where simultaneous implantation can be performed, an implant may assist in maintaining the volume of the sub-antral space [33], but this was not supported in other studies [34–36]. As a result, if the scope of the elevated sinus membrane is not excessive in the square maxillary sinus, it may lead to less periapical bone in the implant at the follow-up visit after six months or even the implant in direct contact with the Schneiderian membrane (Fig. 7), which may damage the sinus membrane, leading to perforation [37] and affect the long-term survival of the implant [38]. It should be noted that in the process of increasing S_bc_ by elevating the membrane, for those maxillary sinuses with an acute angle between the sinus walls or sinus septa and obvious vessels are present, it is also vital to control the angle and strength of the surgical instruments to avoid bleeding and perforation of the Schneiderian membrane [39], or consider using hydraulic pressure [40–42]. For researchers, S_bc_ should be considered as one of the variables when studying the factors influencing osteogenesis of the maxillary sinus lifting surgery, and its value should be recorded and inserted into statistical models or controlled as an irrelevant variable when studying other influencing factors.

In addition, this study corroborates the conclusions of previous studies that V_1_ is significantly positively correlated with V_a_, which means the more graft material, the more it is absorbed [43]. Simultaneous and delayed implant placement results in similar bone augmentation in line with two separate studies with a short-term follow-up of four months after loading and a long-term follow-up of 24 months after surgery [34, 35]. Thus, the choice of implant timing for patients who need sinus floor lift surgery does not affect the osteogenic effect, so clinicians should choose simultaneous or delayed implant methods based on bone mineral density and the overall cortical bone thickness [44].

This study confirmed that bone formation is affected by the contact area between the sub-antral space and surrounding bone after lateral window sinus elevation surgery. Moreover, the contact area between the sub-antral space and the Schneiderian membrane has no effect; thus, it is possible to reject the null hypothesis. However, this study has some limitations, such as the time point at which CBCT images were collected (6 months after surgery) only provides information on prognosis in the short term, and images at follow-up visits may be needed to confirm these results [45]. Furthermore, only one bone graft material (Bio-oss) was used in this study, so the applicability of the findings to other materials needs to be investigated. Lastly, similar to other studies, only imaging methods were used to analyze the effect of contact area on osteogenesis [39, 46], and it is better to use histological studies to further verify the results [47]. Further larger, longer-term studies are required to confirm these results.

## Conclusions

This study confirmed that the size of S_bc_ in lateral window sinus elevation surgery affects osteogenesis after six months of healing, as S_bc_ is positively correlated with V_2_%. Therefore, clinicians should assess the sinus contour type preoperatively to consider whether the range of the sinus membrane elevation should be expanded and the amount of bone graft material increased to avoid excessive collapse of the sub-antral space.

## Data Availability

The datasets used and analyzed during the current study are available from the corresponding author on reasonable request.

## Abbreviations

CBCT: Cone beam computed tomograph
V_1_: Postoperative sub-antral space’s volume
CGF: Concentrated growth factor
S_1_: Postoperative sub-antral space’s surface area
V_2_: Remaining volume after 6 months of healing
S_bc_: Contact area of sub-antral space with maxillary sinus bone
S_mc_: Contact area of sub-antral space with schneiderian membrane
S_j_: The surface area of an imaginary plane, which divides the sub-antral space into upper and lower parts, the upper part contacts the Schneiderian membrane, while the lower part contacts the sinus bone
S_b_: Surface area of the part in contact with bone
S_m_: Surface area of the sub-antral space in contact with the Schneiderian membrane
S_r_: Surface area of the removed lateral bone wall
V_a_: Absorbed volume during healing
V_2_%: Percentage of remaining volume
V_a_%: Percentage of absorbed volume
RBH: Residual bone height
T_0_: Preoperative
T_1_: Immediately after operation
T_2_: 6 Months after operation
